# Drivers' unsafe behaviors in Iran: An investigation in West Azerbaijan

**DOI:** 10.3389/fpubh.2022.815380

**Published:** 2022-12-02

**Authors:** Fatemeh Bakhtari Aghdam, Karim Shaheian, Homayoun Sadeghi-Bazargani, Ahmad Kousha, Koen Ponnet, Mahdieh Abbasalizad Farhangi, Leila Jahangiry

**Affiliations:** ^1^Road Traffic Injury Research Center, Tabriz University of Medical Sciences, Tabriz, Iran; ^2^Department of Health Education and Promotion, Tabriz University of Medical Sciences, Tabriz, Iran; ^3^Faculty of Social Sciences, imec-mict-Ghent University, Ghent, Belgium; ^4^Department of Community Nutrition, Faculty of Nutrition, Tabriz University of Medical Sciences, Tabriz, Iran; ^5^Tabriz Health Services Management Research Center, Tabriz University of Medical Sciences, Tabriz, Iran

**Keywords:** behavior, safety, drives, observational-methods, accident

## Abstract

**Background:**

The present study aims to investigate one of the major causes of traffic accidents: drivers' unsafe behaviors while driving.

**Methods:**

In this cross-sectional study, the behaviors of 946 drivers at traffic lights were observed in the morning, at noon, and in the evening using direct in-field observation. The unsafe behaviors of the drivers included not fastening the seat belt, using a cellphone or handsfree device, smoking, being distracted by a child, talking with passengers, not observing the stop line, eating and drinking, and getting out of the car, letting out a passenger, or arguing with a passenger at the traffic light.

**Results:**

Of the drivers at the traffic light, 60% did not obey the stop line, and 72% did not fasten their seat belt. Also, 13.6% used their cellphones, and 22% talked with passengers. The frequency of the other unsafe behaviors was <3%. For wearing seat belts, drivers aged 41–50 years wore seat belts almost five times more than drivers under 25 years of age (4.94 [2.36–10.320]; *p* < 0.001), and drivers aged 50 years and older were almost three times likelier to wear seat belts than drivers under 25 years of age (2.8 [1.31–6.08]; *p* < 0.001). The results showed that the drivers were significantly likelier to wear seat belts on Saturdays (after the weekend) (0.56 [0.40–0.78]; *p* = 0.001). Regarding using mobile phones while driving, women were twice as likely to use mobile phones as men (2.20 [1.30–3.72]; *p* < 0.001). Drivers aged 26–40 years used mobile phones significantly less than drivers under 25 years of age (0.24 [0.14–0.43]; *p* < 0.001) and drivers aged 41–50 years were significantly less likely to use mobile phones than drivers under 25 years of age (0.19 [1.31–6.08]; *p* < 0.001).

**Conclusion:**

The results showed that the occurrence of wearing a seat belt in Shahin Dej was low. We observed a significant association between wearing a seat belt, age, whether it was Saturday (a day after weekend for Iranians). Additionally, similar associations were observed between using mobile phones and gender, age, and day of the week.

## Introduction

Worldwide, road traffic injuries are among the major general health problems and prominent causes of death worldwide (1). Driving accidents cause nearly 3,300 deaths on a daily basis. Also, 55–136 thousand individuals are injured due to driving accidents around the world every day. In Iran, road traffic injuries are the cause of 16,000 deaths and 335,000 injuries annually (2). Traffic accidents and deaths from high-risk traffic behaviors are among the highest in the world (3).

Various factors contribute to the occurrence of driving accidents. In nearly 90–95% of driving accidents, driver's behaviors are recognized as the main cause of accidents (4). Some simple behavioral changes can lead to desirable outcomes in traffic safety (5). Thus, it is necessary to consider the drivers' behaviors (6, 7).

Some of the driving behaviors that contribute to the occurrence of road traffic injuries include sleepiness, not fastening the seat belt, using hands free, sending messages, high speed, smoking, using cellphones, and talking with passengers. These behaviors increase the probability of driving accidents, and gender and age are also noted as contributing factors (8–11).

By observing 8,240 drivers in Thailand, the prevalence of mobile phone use was reported as 9.9, and 6.5% of drivers used mobile phones while waiting at red traffic lights (12). Unsafe driving behaviors have been observationally investigated in other countries, such as Spain and Saudi Arabia (13, 14). Alghnam et al. reported that more than 13% of drivers used mobile phones while driving, and <34% of them wore seat belts in Riyadh, the capital of Saudi Arabia (14).

In studies conducted on drivers' behaviors, the self-reporting method has been used (15, 16). Self-reporting studies do not reflect the real behavior of drivers. Self-report studies measure general behaviors at different times, and participants think about special times and respond accordingly; however, observational studies are more suitable for investigating road user behavior (17, 18). Several questionnaire-based studies have been conducted in Iran to investigate the prevalence and determinants of unsafe driving behaviors (19–21). Zamani et al. found that individual factors were the most important in the risky driving behavior of taxi drivers (19). A study on the dangerous behavior of drivers in Yazd reported that talking with other passengers (73.6%), consuming foodstuff (42.7%), unfastening the safety belt (38.7%), and talking on cell phones (36.7%) were prevalent in 18–30-year-old drivers (21).

One of the valid methods to observe drivers' behavior is Martínez-Sánchez's method, in which drivers who have stopped behind a traffic light continue their poor driving behavior, even after passing the traffic light (13). In other words, behaviors such as fastening seat belts, smoking, and using cellphones can be done by the drivers before arriving at a traffic light and may continue after passing the traffic light (17).

Direct observation of driving behaviors may provide stronger and more valid documentation of actual risky driving behaviors than self-reported measurements. There are limited studies focusing on high-risk driving behavior through direct observation. Adl et al. directly investigated the behavior of taxi drivers in Tehran (22). They found that the total prevalence of unsafe behaviors of taxi drivers in Tehran was about 52.5, and 80% of all drivers exhibited unsafe behaviors. Additionally, they reported that about 50% of taxi drivers did not use seat belts. It seems that drivers in self-reported measurements exaggerated whether they fasten their seat belts. Therefore, using a direct measurement method to investigate high-risk traffic behaviors may provide actual and accurate estimations of these behaviors.

Since only one observational study was undertaken on taxi drivers, it is not generalizable to other drivers in the Iranian population. Additional studies are warranted to obtain reliable measurements. The present study was designed to observe the unsafe behaviors of drivers stopping behind the stop line at traffic lights in Shahin Dej, a region in northwest Iran. We hypothesized that there is an association between unsafe driving behaviors and other variables, namely, age, gender, and time of the day.

## Methods

### Study design and setting

The present cross-sectional study was conducted on 946 drivers in Shahin Dej, a small city in the southern part of West Azerbaijan Province, a region in the northwestern part of Iran, from June 22 to July 12, 2019. In this city, there were 11 intersections, only 3 of which were equipped with traffic lights. However, there was no surveillance camera at these intersections. Since only three intersections were equipped with traffic lights regarding the traffic volume at these points, all three intersections were included in the study. The drivers of the vehicles that stopped at the traffic lights were randomly included in the study using the convenience sampling method.

### Study process and data gathering

The observations were carried out on weekends and two working days during the study period, namely, on Saturday (the first day of the week in Iran), Tuesday (the middle of the week), and Friday (Iranian weekend), in the morning (7–9), at noon (12–14), and in the evening (17–19) at the selected intersections, based on the inclusion and exclusion criteria.

In the present study, the inclusion criteria included the first five drivers who stopped at the traffic light, in line with Martínez-Sánchez's method (13). After the fifth vehicle, no further observations were carried out. Moreover, three-wheel, two-wheel, and heavy vehicles were excluded from the study.

Five trained observers conducted observations and recorded unsafe traffic behaviors behind a red light in each location using an 11-item checklist. The observers were MSc students working as traffic researchers who received three 60-min training sessions on how to complete the study checklist successfully. Their recordings on the first day of data collection were evaluated by one of the researchers (FBA) in the same location to guarantee quality. The evaluation of the observer's record was continued until they reported the same observations of the target behavior of drivers, and agreement was obtained.

Regarding the results of Beutel et al.'s (23) study with the expectation of 30% for drivers' behaviors as unsafe, *d* = 0.03 and confidence interval = 95%, the sample size was obtained equal to 946. Martínez-Sánchez et al.'s method as a direct in-field observation of drivers' behaviors model was used for data collection. In this method, the behaviors of the drives of the first five vehicles at the traffic light are recorded. In the present work, similarly, the observers stood at the intended intersections, observed the behaviors of the drivers at the traffic light, and recorded their behaviors on a checklist. The behaviors specified in Martinez's study included fastening the seat belt, smoking, and using a cell phone. In this study, other unsafe behaviors, such as arguing with passengers or other drivers, were also included.

The instrument used for data collection included the researcher-made checklist and observation of the drivers' behaviors. The initial checklist had 11 items that were developed by reviewing the existing texts (13, 22, 23). The items of the checklist included fastening the seat belt, using a cellphone (including checking the cell phone, messaging, talking), using handsfree, smoking, being distracted by a child, talking with passengers, not observing the stop line, eating and drinking, getting out of the car, letting out a passenger, and arguing with a passenger.

To examine content validity, the checklist was given to 12 traffic experts (two HSE specialists working in the traffic and transportation office, two psychologists, two sociologists, four health education and behavior specialists, and two epidemiologists).

The content validity index (CVI) was calculated based on three indices: simplicity, relationship, and transparency. Also, the content validity ratio (CVR) was calculated based on the item necessity. Afterward, the necessary changes were applied to the checklist. According to the opinion of the experts, in addition to the behaviors observed in Martinez et al.'s (13) study, it was decided to observe other behaviors, including eating and drinking, disobeying the stop line, arguing with passengers, and getting out of the car. A CVR of 0.81 and a CVI of 0.74 indicated appropriate content validity of the checklist. The checklist consisted of two parts. The first part included the age, gender, and place, time, and day of observation (holiday and working day). The second part involved the observation of the drivers' behaviors with 13 items, including fastening the seat belt, using a cellphone (checking a cell phone, messaging, and talking), using handsfree, smoking, being distracted by a child, talking with passengers, not observing the stop line, eating and drinking, getting out of the car, letting out a passenger, and arguing with a passenger. These items were recorded using the words “Yes” or “No” (Supplementary material).

### Ethical approval

This study was conducted in accordance with the ethical standards of the 1964 Helsinki Declaration. Ethical approval was provided by the Ethical Committee, Tabriz University of Medical Sciences, with reference number IR.TBZMED.REC.1398.1242.

### Analytical strategy

The analyses were carried out using SPSS^®^, version 23.0 (IBM Corporation, Armonk NY, USA). The numbers and percentages were provided for the categorical variables. The normality of distribution for the data was checked by applying the Kolmogorov–Smirnov test. To assess the relationship between driving behavior and the demographic variables, the chi-square test was applied. Multiple binary logistic regressions were used to examine the relationship between independent variables (sex, age, day of the week) and binary outcomes, including Using a seat belt and cellphone use (talking, checking the cell phone, and texting). Adjusted odds ratios and 95% confidence intervals were calculated for all parameters. *P*-values <0.05 were regarded as statistically significant.

## Results

Most of the drivers (70%) were between 25 and 50 years old. More than 90% of the drivers were male. The observations were carried out mostly on the first day of the week (Saturday) and almost equally at three time intervals, including the morning, noon, and evening (Table 1).

**Table 1:** Demographic characteristics of drivers at traffic lights in Shahin De, Iran (N = 946).

**Age**	***N* (%)**
<25	77 (8.1)
26–40	349 (36.9)
41–50	331 (33.9)
>50	199 (21)
**Gender**	
Male	853 (90.2)
Female	93 (9.8)
**During the week**	
Saturday	453 (47.9)
Tuesday	303 (32)
**On weekend**	
Friday	190 (20.1)
**Time of day**	
In the morning	276 (29.2)
At noon	318 (33.6)
In the afternoon	352 (37.2)

Most of the drivers (70%) did not fasten their seat belts. In the age group of 40–50 years old, almost half of the drivers fastened their seat belts, but only a few of the drivers under 40 years old and above 50 years old exhibited this behavior. More than 83% of the observed drivers did not use cellphones, but in the age group of below 40 years, the use of cellphones was significantly higher than the other age groups. Nearly 60% of the observed drivers did not obey the stop line at the traffic lights (Table 2). The frequency of other unsafe behaviors was very low among the observed drivers.

**Table 2:** Behaviors of drivers at traffic lights based on age and gender (N = 946).

	**Total**	**Age *N* (%)**				**Chi-square (df)**	***P*-value**	**Gender**		**Chi-square (df)**	***P*-value**
		**<25**	**25–40**	**41–50**	**>50**			**Men**	**Women**		
Fastened seat belt (yes)	263 (27.8)	9 (11.7)	77 (22.1)	122 (38.0)	55 (20.9)	32.34 (3)	<0.001	229 (26.8)	34 (36.6)	91.4 (1)	0.03
Talking on cell phone (yes)	110 (11.6)	29 (37.7)	41 (11.7)	25 (7.8)	15 (7.5)	58.6 (3)	<0.001	90 (10.6)	20 (21.5)	9.7 (1)	0.003
Writing message (yes)	9 (1)	2 (2.6)	4 (1.1)	2 (0.6)	1 (0.5)	3.14 (3)	0.36	9 (1)	0	0.99 (1)	0.39
Checking cellphone (yes)	26 (2.7)	2 (2.6)	10 (2.9)	13 (4)	1 (0.5)	5.81 (3)	0.12	22 (2.6)	4 (4.3)	0.93 (1)	0.24
Using handsfree (yes)	28 (3)	1 (1.3)	15 (4.3)	9 (2.8)	3 (1.5)	4.4 (3)	0.22	25 (2.9)	3 (3.2)	0.025 (1)	0.53
Eating and drinking (yes)	17 (1.8)	1 (1.3)	3 (0.9)	8 (2.5)	5 (2.5)	3.3 (3)	0.34	13 (1.5)	4 (4.3)	3.66 (1)	0.07
Smoking (yes)	30 (3.2)	2 (2.6)	9 (2.6)	8 (2.5)	11 (5.5)	4.56 (3)	0.20	28 (3.3)	2 (2.2)	0.35 (1)	0.42
Stopping at stop line (yes)	404 (42.7)	35 (45.5)	169 (48.4)	137 (42.7)	63 (31.7)	14.8 (3)	0.002	369 (43.3)	35 (37.6)	1.08 (1)	0.17
Talking with passenger (yes)	206 (21.8)	13 (16.9)	78 (22.3)	82 (25.5)	33 (16.6)	6.97 (3)	0.07	178 (20.9)	18 (30.1)	4.2 (1)	0.03
Picking up pedestrians (yes)	5 (0.5)	0	1 (0.3)	1 (0.3)	3 (1.5)	4.71 (3)	0.19	4 (0.5)	1 (1.1)	0.58 (1)	0.4
Distracted by children (yes)	8 (0.8)	0	2 (0.6)	2 (0.6)	4 (2)	4.37 (3)	0.22	6 (0.7)	2 (2.2)	2.09 (1)	0.18
Getting out of car (yes)	4 (0.4)	0	3 (0.9)	0	1 (0.5)	3.3 (3)	0.34	4 (0.5)	0	0.43 (1)	0.66
Arguing (yes)	1 (0.1)	1 (1.3)	0	0	0	11.3 (3)	0.01	1 (0.1)	0	0.109 (1)	0.9

Less than 10% of the observed drivers were female. The female drivers used seat belts significantly more than the male drivers. Also, female drivers (21.5%) talked on their cellphone significantly more than male drivers (10.5%). Moreover, the female drivers talked with their passengers more than the male drivers did (Table 2).

The use of a seat belt on the first day of the week was significantly higher than on the middle day of the week (22.5 vs. 32.5%). However, cell phone use and smoking on holidays occurred more often than on working days. On holidays, the drivers stopped behind the stop line significantly more than the other days of the week. No significant difference was observed between the behaviors at different times during the day, except the stop line item. More than 50% of the drivers did not stop behind the stop line in the morning, which was significantly higher compared to noon (Table 3). The association of wearing a seat belt and mobile use with gender, age, and days of the week was assessed using multiple binary logistic analyses (see Table 4). For wearing seat belts, drivers aged 41–50 years wore seat belts almost five times more than drivers under 25 years of age (4.94 [2.36–10.320]; *p* < 0.001), and drivers aged 50 years and older were almost three times likelier to wear seat belts than drivers under 25 years of age (2.8 [1.31–6.08]; *p* < 0.001). The results showed that the drivers were significantly likelier to wear seat belts on Saturdays than Tuesdays (0.56 [0.40–0.78]; *p* = 0.001). For using mobile phones while driving, women were twice more likely to use mobile phones than men (2.20 [1.30–3.72]; *p* < 0.001). Drivers aged 26–40 years used mobile phones significantly less than drivers under 25 years of age (0.24 [0.14–0.43]; *p* < 0.001) and drivers aged 41–50 years were significantly less likely to use mobile phones than drivers under 25 years of age (0.19 [1.31–6.08]; *p* < 0.001). The results showed that the drivers were significantly likelier to use mobile phones on Fridays (weekends) than Tuesdays (1.82 [0.67–1.49]; *p* = 0.02).

**Table 3:** Behaviors of drivers at traffic lights based on day of the week and time of day (N = 946).

**Behaviors**	**Total**	**Week *N* (%)**				**Time of the day *N* (%)**			
		Saturday^*^	Tuesday^***^	Friday^**^	***P*-value**	**Morning**	**Noon**	**Afternoon**	***P*-value**
Fastening seat belt (yes)	263 (27.8)	102 (22.5)	99 (32.7)	62 (32.6)	0.002	76 (27.5)	97 (30.5)	90 (25.6)	0.360
Talking on cellphone (yes)	110 (11.6)	52 (11.5)	31 (10.2)	27 (14.2)	0.403	36 (13.0)	41 (12.9)	33 (9.4)	0.250
Writing message (yes)	9 (0.1)	1 (0.2)	3 (1.0)	5 (2.6)	0.016	5 (1.8)	2 (0.6)	2 (0.6)	0.21
Checking cellphone (yes)	26 (2.7)	11 (2.4)	5 (1.7)	10 (5.3)	0.049	8 (2.9)	9 (2.8)	9 (2.6)	0.96
Using handsfree (yes)	28 (3)	12 (2.6)	7 (2.3)	9 (4.7)	0.26	10 (3.6)	8 (2.5)	10 (2.8)	0.71
Eating and drinking (yes)	17 (1.9)	3 (0.7)	7 (2.3)	7 (3.7)	0.02	3 (1.1)	8 (47.1)	6 (2.5)	0.42
Smoking (yes)	30 (3.2)	8 (1.8)	4 (1.3)	18 (9.5)	0.001	13 (4.7)	13 (4.1)	4 (1.1)	0.02
Stopping at the stop line (yes)	404 (42.7)	241 (53.2)	137 (45.2)	26 (13.7)	0.001	147 (53.3)	113 (35.5)	144 (40.9)	0.001
Talking with passenger (yes)	206 (21.8)	105 (23.2)	72 (23.8)	29 (15.3)	0.051	51 (18.5)	70 (22)	85 (24.1)	0.23
Picking up pedestrians (yes)	5 (0.5)	3 (0.7)	0	2 (1.1)	0.25	0	1 (0.3)	4 (1.1)	0.12
Distracted by children (yes)	8 (0.8)	1 (0.2)	0	7 (3.7)	0.001	2 (0.7)	3 (0.9)	3 (0.9)	0.95
Getting out of the car (yes)	4 (0.4)	4 (0.9)	0	0	0.11	2 (0.7)	0	2 (0.6)	0.34
Arguing (yes)	1 (0.1)	1 (0.2)	0	0	0.58	0	1 (0.3)	0	0.37

* Saturday is the first day of the week in Iran.

** Friday is like Sunday in other countries.

*** Tuesday is the middle of the week in Iran.

**Table 4:** Binary logistic regression model of the association between wearing seat belts and cell phone use among drivers.

	**Wearing a seat belt**		**Mobile phone use**	
	**OR (CI 95%)**	***P*-value**	**OR (CI 95%)**	***P*-value**
**Gender**				
Male	Ref	Ref	Ref	Ref
Female	Not included	Not included	2.20 (1.30–3.72)	<0.001
**Age**				
<25	Ref	Ref	Ref	Ref
26–40	2.1 (1.04–4.62)	0.39	0.24 (0.14–0.43)	<0.001
41–50	4.94 (2.36–10.32)	<0.001	0.19 (0.11–0.34)	<0.001
>50	2.8 (1.31–6.08)	<0.001	0.13 (0.06–0.26)	<0.001
**Days of the week**				
Tuesday	Ref	Ref	Ref	Ref
Saturday	0.56 (0.40–0.78)	0.001	1.09 (0.69–1.71)	0.69
Weekend (Friday)	1.00 (0.67–1.49)	0.98	1.82 (1.09–3.03)	0.02

## Discussion

This observational study was conducted to investigate the unsafe behaviors of drivers at traffic lights in Shahin Dej, Iran. The obtained results indicated that more than 70% of the drivers did not fasten their seat belts, although fastening the seat belt is obligatory for drivers and all passengers of all vehicles on all roads in Iran. In terms of sociodemographic factors related to seat belt use, there was a significant association between age and the day of the week. Additionally, similar associations were observed between using mobile phones and gender, age, and day of the week.

Different studies in Iran have shown that more than 50% of drivers (22, 24) did not use seat belts. In our study, the findings were about 20% higher. This discrepancy might perhaps be explained by the observational design of our research. In fact, the present observational study measured actual unsafe behavior in a real environment and can be an indicator of the exact behavior. However, self-report studies may have over/underestimated real safety behavior.

Not fastening the seat belt is one of the main causes of death in traffic accidents, accounting for nearly 63% of deaths. However, studies have shown that fastening the seat belt can reduce the risk of car accidents by up to 38–46% for drivers and 45% for passengers on the front seat (4). In traffic accidents, the major cause of driver and passenger death is a head strike, which can be reduced by fastening the seat belt (25). In the national traffic safety education program, education about the reasons for using the seat belt and its importance in reducing the risk of accidents is necessary content for all target groups (25).

Similarly, a study in Saudi Arabia showed that 34% of drivers used seat belts (14). On the other hand, 94% of drivers in the US use seat belts while driving (26). Based on the findings of this study, the low use of seat belts, despite the rules and fines for unfastened seat belts, is due to the lack of contingent vision about the probable lethal consequences of unfastened seat belts, forgetting to fasten the seat belt, feeling uncomfortable, and wrinkling of clothes (27). Accordingly, it is necessary to provide behavioral actions and interventions, such as training campaigns, to improve society's knowledge and awareness in this regard.

In the present study, women used seat belts significantly more often than men (10%). Similarly, other studies have shown similar results, perhaps since women may care more about their safety and health during driving than men (28–30). Thus, it is necessary to prioritize male drivers for educational and interventional programs about seat belt use.

According to the regression analyses, drivers aged 41–50 years old use seat belts almost five times more often than young drivers. It is likely that these drivers have been involved in traffic accidents and have a better understanding of safety, traffic citations, and traffic laws (31). They may also be more experienced and make more reasonable decisions while driving (32). The results showed that the use of a seat belt on Saturday was 44% less than on Tuesday and Friday. This result was consistent with Torkamannejad et al.'s study's, which reported that the drivers used seat belts more often than on working days (32). However, the result was inconsistent with another study in Nigeria, which found that seat belt use was higher on Monday, their first working day, than on Saturday and Sunday (33).

According to the findings of our study, 16.6% of the drivers used cellphones, wrote messages, checked their cellphones, and used hands free while driving. Consistent with the present study, observing driver behavior in Riyadh indicated that 13.8% of drivers used a cellphone while driving (14). Although forbidden, cellphone use is common among drivers (34).

Using cellphones and other electronic devices while driving is the main cause of distraction among drivers (22, 35), which can increase the probability of accidents by 3–6.5% and have negative effects on driving performance (34). It also accounts for 25% of all accidents, including those ranging from having slight damage to accidents with mortalities (36).

Among the functions of cellphones, sending SMS messages while driving causes maximum distraction (37). Sending messages requires mental engagement and, thus, the driver takes his/her eyes off the road much more than when s/he is talking by cellphone; as a result, the negative effects of sending a message are more intense than those of receiving a message (38, 39). Studies have shown that sending or receiving text messages while driving can increase the probability of an accident 23 times (40). To reduce cellphone use while driving, perhaps reinforcing and supervising the proper execution of rules and regulations, and these regulations could be a good strategy. Also, education *via* a simulator can play a significant role in this regard (41), as the use of modern technology might be helpful. For instance, a phone call can be automatically disconnected or a messaging function deactivated in a moving vehicle. Of course, this requires consideration of all aspects of the issue.

In the present study, a significant relationship was observed between the use of cellphones and age group and gender. Accordingly, women used cellphones twice as often as men. Studies have shown that women use cell phones more to talk and text, while men use them for networking, game playing, and other applications (42, 43). There is a need to show and educate women about the dangers of simultaneously driving and using mobile phones, which could also be done with simulations.

A strong association between mobile phone use and young age was found in the multivariate model. According to the results, mobile phone use decreased with age. Several studies (44–46) have shown that young drivers used the cellphone while driving more than other groups, which can be because young individuals use modern technologies much more than other age groups (46). Moreover, young drivers are likelier to send messages while driving (47).

According to the findings, 42.2% of the observed drivers did not obey the stop line at red traffic lights, regardless of their obligation to do so. The observers attributed this to the drivers' distraction, their hurry to pass the traffic light, the age of the crosswalks, and perhaps the lower visibility of the stop lines.

Furthermore, based on the findings of the present study, there was a significant relationship between the time of driving and violation of the stop line, with such behaviors occurring mostly in the morning. Similarly, two studies indicated a significant relationship between drivers' unsafe behaviors and the time of observation of their behaviors, but the observed drivers showed unsafe behaviors mostly in the evenings (20, 22).

Perhaps in our study, drivers were in a hurry in the mornings to reach their workplaces and did not want to wait behind the stop lines. Moreover, the results of the present study showed that obeying the stop line on the holidays was much more than that on other days.

The results showed that mobile phone use was 1.8 times higher on the weekend than in the middle of the week. According to the findings, 21.8% of the drivers talked with passengers. In this regard, there was a significant relationship between the driver's gender and talking with passengers, with female drivers talking with passengers more often than male drivers. Conversation between a driver and passengers is one of the main causes of a driver's distraction (48, 49). It seems necessary to provide knowledge and education directed toward women.

One of the strong points of the present work was that the real behaviors of the drivers in real environments were recorded exactly. The observers stood at points where they could observe and record the drivers' behaviors without attracting their attention; thus, the results of this study were more realistic than those of the self-reporting studies. This study was the first observational study conducted in the northwestern part of Iran in which drivers' behaviors were investigated objectively.

One of the limitations of the present study was that, regarding the observational nature of the study, many characteristics of the drivers, such as educational level, marital status, economic status, and exact age, which could influence their behavior, were not possible to investigate. Moreover, the observers were likely to make mistakes in terms of recording the approximate age of the participants, since the drivers' ages were recorded only based on superficial features. The drivers in this study may not be representative of people driving in other Iranian communities. Shahind Dej, our study setting, is a small city, and the number of female drivers included in this study was limited.

## Conclusion

The results show that seat belt usage in Shahin Dej is low. In terms of sociodemographic factors related to wearing a seat belt, there was a significant association between age and days of the week. Additionally, similar associations were observed between cell phone use and gender, age, and days of the week. This observational study was a good resource for showing unsafe behaviors among drivers, such as not fastening the seat belt, using a cellphone, talking with passengers, and disobeying the stop line.

## Data availability statement

The original contributions presented in the study are included in the article/Supplementary material, further inquiries can be directed to the corresponding author.

## Ethics statement

The studies involving human participants were reviewed and approved by Tabriz University of Medical sciences. The patients/participants provided their written informed consent to participate in this study.

## Author contributions

FB and HS-B were responsible for the study design. LJ did the analyses. LJ, HS-B, AK, KS, and FB were responsible for data interpretation. LJ, KP, MAF, and FB drafted the manuscript. All authors have read and approved the final manuscript.

## Funding

This work was supported by Tabriz University of Medical Sciences.

## Conflict of interest

The authors declare that the research was conducted in the absence of any commercial or financial relationships that could be construed as a potential conflict of interest.

## Publisher's note

All claims expressed in this article are solely those of the authors and do not necessarily represent those of their affiliated organizations, or those of the publisher, the editors and the reviewers. Any product that may be evaluated in this article, or claim that may be made by its manufacturer, is not guaranteed or endorsed by the publisher.

## Abbreviations

CVI: Content validity index
CVR: Content validity ratio.
