# Supplementary material for: Drivers' unsafe behaviors in Iran: An investigation in West Azerbaijan
Source: Front Public Health. 2022 Dec 2;10:815380. doi: 10.3389/fpubh.2022.815380 (PMC9755251; doi:10.3389/fpubh.2022.815380)
Supplement: Supplementary file 1 [file Table_1.docx]

| Variable | Yes | No |
| --- | --- | --- |
| Driver’s sex |  |  |
| Man |  |  |
| Woman |  |  |
| Age (Years) |  |  |
| <25 |  |  |
| 25-40 |  |  |
| 41-50 |  |  |
| > 50 |  |  |
| Place of observation (name of intersection) |  | |
| Day of observation |  | |
| Saturday (First day of week) |  |  |
| Tuesday (middle day of the week) |  |  |
| Friday (weekend) |  |  |
| Time of observation |  |  |
| In the morning |  |  |
| At noon |  |  |
| In the afternoon |  |  |
| Fastening seat belt |  |  |
| Using a cellphone |  |  |
| Checking cell phone |  |  |
| Messaging |  |  |
| Talking |  |  |
| Using handsfree |  |  |
| Smoking |  |  |
| Distracting by a child |  |  |
| Talking with passengers |  |  |
| Not observing the stop line |  |  |
| Eating and drinking |  |  |
| Getting off the car at the traffic light |  |  |
| Getting off the passenger at the traffic light |  |  |
| Arguing with the passenger at traffic light |  |  |

**Drivers' unsafe behaviors in Iran: a check list for recording observations**
